# Home Sampling for Sexually Transmitted Infections and HIV in Men Who Have Sex with Men: A Prospective Observational Study

**DOI:** 10.1371/journal.pone.0120810

**Published:** 2015-04-07

**Authors:** Martin Fisher, Sonali Wayal, Helen Smith, Carrie Llewellyn, Sarah Alexander, Catherine Ison, John V Parry, Garth Singleton, Nicky Perry, Daniel Richardson, on behalf of the Brighton Home Sampling Kit Project Steering Group

**Affiliations:** 1 Department of Genitourinary/HIV Medicine, Brighton and Sussex University Hospitals NHS Trust, Brighton, United Kingdom; 2 Division of Primary Care and Public Health, Brighton and Sussex Medical School, Falmer, United Kingdom; 3 Public Health England, Sexually Transmitted Bacteria Reference Laboratory and Virus Reference Department, London, United Kingdom; 4 Institute of Development Studies, University of Sussex, Brighton, United Kingdom; National HIV and Retrovirology Laboratories, CANADA

## Abstract

To determine uptake of home sampling kit (HSK) for STI/HIV compared to clinic-based testing, whether the availability of HSK would increase STI testing rates amongst HIV infected MSM, and those attending a community-based HIV testing clinic compared to historical control. Prospective observational study in three facilities providing STI/HIV testing services in Brighton, UK was conducted. Adult MSM attending/contacting a GUM clinic requesting an STI screen (group 1), HIV infected MSM attending routine outpatient clinic (group 2), and MSM attending a community-based rapid HIV testing service (group 3) were eligible. Participants were required to have no symptomatology consistent with STI and known to be immune to hepatitis A and B (group 1). Eligible men were offered a HSK to obtain self-collected specimens as an alternative to routine testing. HSK uptake compared to conventional clinic-based STI/HIV testing in group 1, increase in STI testing rates due to availability of HSK compared to historical controls in group 2 and 3, and HSK return rates in all settings were calculated. Among the 128 eligible men in group 1, HSK acceptance was higher (62.5% (95% CI: 53.5–70.9)) compared to GUM clinic-based testing (37.5% (95% CI: 29.1–46.5)), (p = 0.0004). Two thirds of eligible MSM offered an HSK in all three groups accepted it, but HSK return rates varied (highest in group 1, 77.5%, lowest in group 3, 16%). HSK for HIV testing was acceptable to 81% of men in group 1. Compared to historical controls, availability of HSK increased the proportion of MSM testing for STIs in group 2 but not in group 3. HSK for STI/HIV offers an alternative to conventional clinic-based testing for MSM seeking STI screening. It significantly increases STI testing uptake in HIV infected MSM. HSK could be considered as an adjunct to clinic-based services to further improve STI/HIV testing in MSM.

## Introduction

In the UK men who have sex with men (MSM) are disproportionately affected with HIV and sexually transmitted infections (STI). The rates of STIs and HIV among MSM are increasing, and the uptake of STI and HIV testing in conventional clinical settings is suboptimal [1]. The prevalence of undiagnosed HIV and late HIV diagnosis among MSM continues to be high [1,2]. The introduction of national targets for improved access to Genitourinary Medicine (GUM) services [3] has increased the availability of testing in the clinical environment [4]. National HIV testing guidance and community-based organisations have increasingly advocated the broadening of STI/HIV testing to non-clinical sites to increase HIV testing uptake and reduce the burden of undiagnosed HIV [5,6]. There is a need to develop novel approaches to increase HIV testing amongst MSM. Furthermore, since it is well established that the presence of STI facilitates HIV transmission and acquisition [7–9] there is a need to optimise STI testing in MSM.

Home sampling/testing may be one such initiative to meet these national aims. As part of England’s National Chlamydia Screening Programme (NCSP) self sampling has been widely used to test for *Chlamydia trachomatis* (CT). This programme has primarily targeted females, and almost exclusively a heterosexual population [10]. Our research group has confirmed that home sampling is an acceptable alternative to conventional testing in MSM [11,12], rectal and oropharyngeal self-sampling is acceptable and feasible [13], and performs as well as conventional nurse-taken diagnostic sampling [14].

The objectives of this study were to determine uptake of home sampling for HIV and STIs compared to conventional clinic-based testing, and to determine whether the availability of home sampling would increase STI testing amongst HIV infected MSM and those attending a community-based HIV testing service compared to historical controls. We hypothesised that the uptake of home sampling for sexual health screening would be higher compared to conventional testing among asymptomatic MSM accessing a GUM clinic. We also hypothesised that offering home sampling for STI testing to HIV positive MSM attending an HIV outpatient clinic and to MSM attending a community-based HIV testing service would increase rates of STI testing.

## Methods

### Design

The study was a prospective observational study evaluating the uptake of home sampling for STI/HIV, and examine whether the availability of home sampling would increase STI testing rates amongst HIV infected MSM and those attending a community-based HIV testing clinic compared to a historical control (i.e. the comparator was testing rates in the corresponding calendar period in the previous year).

### Participants

The study was conducted between February and September 2008 in three sites providing STI and/or HIV testing services in Brighton, UK. Eligible individuals were HIV negative (by self–report) MSM attending in person or contacting the GUM clinic via telephone requesting an STI screen (group 1), MSM with HIV infection attending the HIV outpatient clinic for routine outpatient follow-up (group 2), and MSM attending a rapid HIV testing service provided by the GUM clinic in a community-based organisation and (group 3). Participants were required to have no symptomatology consistent with STI, be 18 years or over, and were known to be immune to hepatitis A and B [previously vaccinated or with documented evidence of natural immunity] (group 1), and were attending for routine HIV follow-up (group 2) or community HIV testing (group 3). Eligibility for the study for group 1 was determined by the reception staff trained in study procedures by the study coordinator and was determined by the clinical staff for groups 2 and 3.

### Procedures

**Fig 1:**
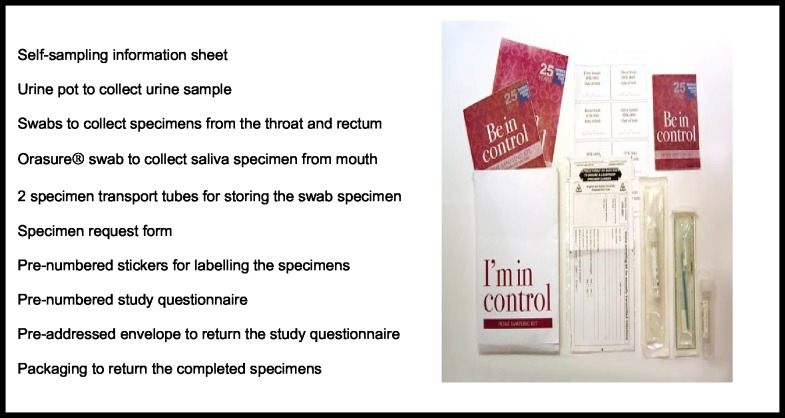
Contents of the home sampling kits.

Eligible men in groups 1 who were seeking STI/HIV testing and group 2 who were seeking regular HIV care in person/contacting the clinic via telephone were offered a home sampling kit (HSK) to obtain self-collected specimens for STI and HIV (group 1) and for STI (group 2) as an alternative to routine testing in the GUM clinic. Group 3 men attending the community service for HIV testing were offered a HSK, for STI only, if their rapid HIV test result was negative. Those opting for home self-sampling were given (or posted in case of group 1) an HSK (Fig. 1) containing a urine pot, throat swab, rectal swab, oral fluid collection device (Orasure), and a participant information sheet which had been developed with patients during an earlier phase of the study [11]. Participants in Group 1 were asked to state on the specimen request form if they wished to have a HIV test as part of their HSK screen. HSKs also contained a pre-numbered questionnaire and an addressed envelope to return the study questionnaire to the research team. All participants were asked to state their preferred method for receiving test results (telephone, letter, or email). Participants from all groups were asked to return the self-collected specimens in person to the GUM or HIV clinic. In the event of non-return one reminder to return the HSK was sent prior to the end of the study period using the participants preferred method of contact specified in the request form. Treatment including partner notification was performed according to standard clinic protocols. The clinic’s contemporaneous policy of ‘no news is good news’ was followed in the context of negative results, unless participants had specifically requested otherwise.

We maintained a prospective record of the total number of eligible men attending the study sites during the study period. Data on the effect of HSK on STI testing rates during the historic control period (the same calendar period as the study period in the previous year, i.e. February-September 2007) for the HIV clinic cohort were extracted from the HIV clinic database which includes data on STI screening, and self-reported STI testing rates in the community cohort were extracted from the clinic records by the clinic staff. The clinic procedures for STI/HIV testing were unchanged between study and historic control periods. HSK decliners in Group 1 completed a brief questionnaire about socio-demographics and preferences regarding STI testing services and men in other groups were asked to give their reasons for declining a HSK.

### Ethics Statement

This study including the consent procedures was approved by the Brighton East Research Ethics Committee (Reference05/Q1907/127). Consent for study participation was implicit based upon the patient choosing to use an HSK and its subsequent return. Thereby, formal written informed consent at the time of recruitment was not obtained. As described previously, HIV testing was performed only for those who had explicitly consented to be tested for HIV on the specimen request form.

### Specimen Collection and Analysis

All HSK specimens were analysed in the Sexually Transmitted Infection Bacterial Reference Unit and Virus Reference Department at Public Health England, Colindale, London, UK. Urine, rectal and pharyngeal swabs were tested for *CT* and *Neisseria gonorrhoea* (GC) using the Aptima Combo 2 assay (AC2). Oral fluid specimen were tested for syphilis (ICE EIA) and if applicable, for HIV antibodies employing an EIA method (GACELISA) [15]. Full details of the testing methodology for CT and GC utilised in this study have been described elsewhere [14].

### Sample size and statistical methods

From our earlier work, which showed that approximately 80% of MSM accessing a GUM service for STI testing would prefer a HSK to conventional testing [13], we estimated a requirement to recruit a minimum sample of 85 asymptomatic MSM attending the GUM clinic in order to detect a difference in acceptability with 80% power and significance of 0.05. For the HIV clinic attendees and for the community setting a minimum sample size of 260 and 140 were required respectively to detect a 10% increase in STI testing rates compared to rates in a historical control period. In the same calendar period in the previous year the STI testing rates were 17% in the community and 13% in the clinic.

The main study outcomes were: uptake of HSK compared to conventional clinic-based testing for STI/HIV in group 1, increase in rates of STI testing due to availability of HSK in groups 2 and 3 compared to historical controls. HSK uptake and return rates between the 3 groups were compared. HSK uptake and return rate for each study site was estimated as the proportion of the total number of eligible participants who accepted a HSK and returned the specimens. STATA v.10 was used for data management and analysis. One sample binomial test was conducted to test the uptake of HSK compared to conventional testing in group 1. χ^2^ test was used to compare STI testing uptake rates due to the availability of HSKs during the study period compared to historical controls (groups 2 and 3), and HSK uptake and return rates between the three groups.

## Results

### Overall HSK uptake and return

**Fig 2:**
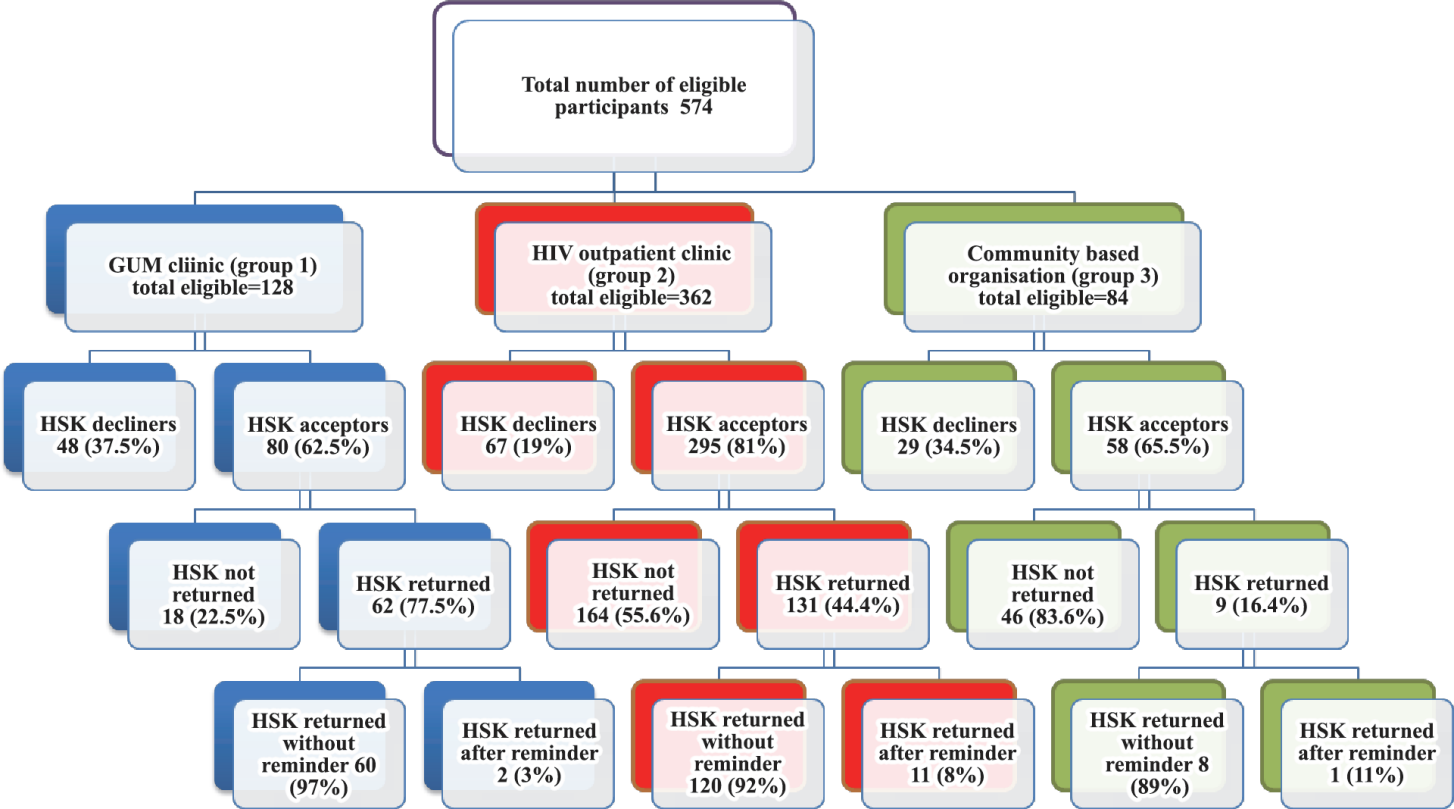
HSK uptake and return in all the study sites.

A total of 574 eligible MSM were offered a HSK in the study period, of whom 433 (75%) accepted. The acceptance rates by testing site are given in Fig. 2. The uptake of HSK by study site differed significantly (χ^2^ 22.8; p<0.001). Uptake was highest amongst those attending the HIV outpatient clinic (group 2; 81%), and was comparatively lower in the GUM clinic (group 1; 63%) and community based organisation (group 3; 66%). Overall, 47% (202/433) HSKs were returned. Among those who had accepted an HSK, there was a significant difference in the return rate by study site (χ^2^ 51.4; p<0.001). The return rate was highest amongst those attending the GUM clinic (group 1; 78%), followed by HIV outpatient clinic (group 2; 44%), and lowest amongst participants from the community based organisation (group 3; 16%).

### Participant characteristics

Study questionnaires were returned by a high proportion of HSK users (92% GUM clinic, 84% HIV outpatients and 100% of HSK users in the community). The median age of participants was 42 years (IQR: 34–48), 87% were white British (Table 1). The majority of men self-identified as gay (98%), were educated beyond secondary school (87%) and 66% were employed. The majority had been sexually active in the last 3 months (94%). Approximately 14% of men in group 1 and 27% of men in group 2 reported engaging in unprotected insertive or receptive anal sex in the last three months. Over a third (37%) had not tested for a STI in the last year.

**Table 1:** Socio-demographic profile, STI testing and sexual behaviour of HSK users (with returned questionnaires).

***Factor***	***GUM***	***HIV Clinic***	***Community***	***Overall***
	***Clinic***		***Organisation***	
	***[%]***	***[%]***	***[%]***	***[%]***
	***N = 57***	***N = 110***	***N = 9***	***N = 176***
**Median Age [in years] [IQR]**	33 [25–44]	44 [38–50]	41 [30–45]	42 [34–48]
**Age Range [in years]**	20–60	20–62	26–52	20–62
**Age distribution**				
18–30	40.4	6.4	22.2	18.2
31–40	26.3	24.5	22.2	25.0
41–50	22.8	46.4	44.4	38.6
>50	10.5	22.7	11.1	18.2
**Sexuality**				
Homosexual	96.5	99.0	100	98.3
**Ethnicity**				
White British	84.2	88.0	88.9	86.9
**Education [n = 171]**				
No formal education	1.8	8.6	0	5.8
A levels/GCSE/NVQ	49.1	50.5	44.4	49.7
Degree and above	45.6	30.5	55.6	36.8
**Employment**				
Working	80.7	57.4	66.7	65.5
**Tested for STI in last year**				
No	37.9	37.3	33.3	36.7
**Had sex in last 3 months**				
Yes	98.3	92.3	78.0	93.5
**Had unprotected insertive anal sex in the last 3 months**				
Yes	14.8	26.6	0.0	21.7
**Had unprotected receptive anal sex in the last 3 months**				
Yes	13.0	30.0	0.0	23.4

### Uptake of HSK compared to clinic-based testing

As hypothesised there was a greater acceptance of HSK (62.5% (95% CI: 53.5–70.9)) compared to conventional GUM clinic-based testing (37.5% (95% CI: 29.1–46.5)) among men in group 1 (p = 0.0004). The uptake of HIV testing amongst these HSK users was 81% (n = 50/62) with the median interval since last HIV test being 9 months (range: 1–186). There were no new HIV diagnoses among MSM in group 1 (compared to one new diagnosis among men who opted for conventional GUM clinic-based testing).

### Impact of HSK on STI testing in HIV and community clinics

The overall STI testing rate in the MSM HIV outpatient clinic cohort increased from 13% (139/1086) in the same calendar period in the previous year to 19% (220/1164; 131 from HSK returners) during the study period (χ^2^ 12.3; p<0.001). The STI testing rates in the MSM cohort attending the community based organisation was unchanged between the two study periods, 17% (21 of 126) in the control period and 18% (15 out of 84) in the study period; 9 of whom were HSK returners (χ^2^ 1.665; p = 0.19).

### Identification of STI

The overall STI prevalence in HSK returners was 13% (26/202). The prevalence was 9% (18/202) for CT (13 rectal, 3 urethral, 2 pharyngeal), GC 2.5% (5/202; 3 pharyngeal, 1 urethral, 1 rectal), and 1.5% (3/202) for newly diagnosed syphilis (n = 3). Prevalence rates were similar for each recruiting site: 13%, (8/62) for the GUM clinic (group 1), 13% (17/128) for the HIV clinic (group 2), and 11% (1/9) for the community clinic (group 3).

### HSK decliners: socio-demographic profile and STI testing behaviour

Based on data collected from 30 of the 48 (63%) decliners in the GUM clinic (group 1) we found no significant differences between men who accepted or declined an HSK in age (median 33 years vs. 35; p = 0.75), STI testing in last year (62% vs. 62.5%; p = 1.00), willingness to wait one day for a clinic appointment (8.6% vs. 8.3%; p = 0.93), or importance of accuracy of test results (95% vs. 98%; p = 0.95). Men in the HIV outpatient clinic declined an HSK because of being in a monogamous relationship (13%), not being sexually active since last STI screen (54%), and/or recent STI screen (40%). Men in the community based organisation surprisingly expressed preference for conventional GUM clinic based STI testing (38%). The commonest reason for declining an HSK in this group was that eligible subjects considered they did not need an STI test (46%), this was either because they had tested for STI recently, or were in monogamous relationship but were testing for HIV because their partner was HIV positive.

## Discussion

In this prospective observational study, we have demonstrated that overall the uptake of home sampling was high amongst MSM attending the GUM and HIV outpatient clinics, and a community-based HIV testing service with acceptance by two-thirds of those offered although return rates varied and were highest among men who actively sought STI testing (group 1). Given that HSKs were both acceptable and likely to be returned, HSK can be offered as a viable alternative to GUM clinic based STI/HIV testing to asymptomatic MSM contacting GUM clinics for an STI screen.

Home sampling for HIV was acceptable to most (81%) MSM in group 1. No new HIV diagnoses were made which is not unexpected given the short duration of our study, and the sample size of our study population. The high rate of acceptability of home self-sampling for HIV suggests that HSK can be a viable method to increase the opportunities of HIV testing through diverse methods as emphasised in the National HIV testing policy and guidelines. Indeed, given that HIV positive status was not a stated exclusion, some of the HIV test non-returners may already have been HIV positive and therefore the acceptability may be even higher than our figures suggest. No new HIV diagnoses were made which is not unexpected given the short duration of our study, and the sample size of our study population.

Since the completion of this study, in the UK self-sampling HIV postal kits are being offered by community based charitable organisations as well as GUM clinics. From April 2014 home self-testing for HIV using point-of-care diagnostic tests has been legalised in the UK. Our study data suggests that for MSM home sampling for HIV is likely to be acceptable; however further evidence regarding the acceptability of home self-testing for HIV, and the effectiveness of home sampling and home testing for HIV on reducing the burden of undiagnosed HIV and increasing the frequency of HIV testing among at-risk MSM is needed.

In the non-GUM settings, although uptake of HSKs was high among MSM attending the HIV outpatient clinic and community-based organisation, return rates were lower compared to group 1. Nevertheless, a high proportion of HIV positive MSM who used the HSK for STI testing had not tested for STI in the last year. When compared to the historical control period, during the study period the availability of HSK increased the proportion of HIV positive MSM testing for STIs in the HIV outpatient clinic group 2. The Health Select Committee on HIV/AIDS has emphasised the significance of STI control for HIV prevention [16]. National guidelines recommend an annual full sexual health screen for people living with HIV regardless of their sexual history [17]. STI testing among HIV positive persons on antiretroviral treatment (ART) is also vital for the success of ART for HIV prevention [18]. These data suggest that offering HSK can enhance STI testing in this group. HSK for STI testing can especially be offered in HIV outpatient clinics where STI testing service is not an integral part of HIV care and treatment service. There was no change in the rate of STI testing observed among men attending the community based organisation suggesting the need for further research to understand barriers to and acceptable methods for STI testing among men accessing services in these settings.

The rates of STI detection amongst those sampling at home was high (13%) and similar to rates seen in asymptomatic MSM attending the GUM clinic in the same time period with a similar distribution of diagnoses of CT, GC and syphilis (8%, 4%, and 1% respectively). Given this similar prevalence rate and the increased uptake of STI testing by the offer of HSKs, we propose that novel strategies such as the offer of home sampling in addition to conventional clinic-based testing is likely to facilitate the recognition of undiagnosed STIs which might otherwise remain undiagnosed.

This is one of the first research studies to evaluate the use of home sampling for STIs and HIV in MSM, a key group disproportionately affected by both STIs and HIV and specifically targeted for novel testing strategies. Our findings are consistent with those of a subsequent study [19] evaluating home sampling in MSM, in demonstrating high rates of acceptability and STI diagnosis. Limitations of the study included the failure to reach the initial planned sample size in the HIV clinic and community based organisation as the HSK return rate in these settings was lower than originally anticipated. It appears that the return rates were much lower when the primary aim of the encounter was not for an STI test but rather a routine outpatient visit or specifically for HIV testing. This low return rate is consistent with that observed in studies in heterosexual communities where home sampling kits are obtained outside the clinical setting [20,21]. The requirement on individuals to return the completed kits in person may have further exaggerated the low return rate we observed. Given that free home HIV sampling kits can now be ordered online from some GUM clinics and community based organisations in the UK, further research and service evaluation is needed to understand if offering postal delivery and return enhances HSK return rates. Since this was a prospective observational study and not a randomised controlled study (randomising potential GUM clinic attenders to the offer of an HSK or not) we are unable to conclude whether the availability of HSKs increase overall rates of STI testing, but can conclude that the offer is acceptable to the majority of MSM seeking STI tests. Also further research among those not returning HSKs is needed to understand their reasons for not doing do. Secondly, the number of eligible subjects from the GUM clinic was much lower than originally anticipated due to a combination of the requirement to be asymptomatic, and the need to have a documented immunity to hepatitis A and B infections. Future evaluation of home sampling should therefore include technologies for hepatitis virus evaluation and should also include the testing of individuals symptomatic of STI if access to conventional GUM services is not possible within a suitable time-frame. Thirdly, this study was conceived at a time when access to GUM services was relatively poor. By the time the study was implemented, a government target of 48-hour access to GUM services was in place and so motivation for opting for home sampling may have been lower than previously anticipated.

A further limitation is that this study was conducted in a single geographical area where uptake of research in GUM/HIV clinics has been traditionally high. Therefore future studies should evaluate acceptability of HSK in other geographical areas. However, since the completion of the study, the routine offer of home sampling for HIV by some clinics throughout the UK suggests this is unlikely to be a significant limitation.

This study serves as a “proof of concept” that home sampling for STI and HIV is acceptable to a significant proportion of MSM and may therefore enable choice and broaden access to testing for both STI and HIV in this target group. Future research should address the potential impact of such increased access to testing on incidence rates of STI in this population and rates of undiagnosed or late presentation of HIV infection. Indeed, a study conducted by Elliot and colleagues has shown that offering home sampling for HIV to high-risk MSM via social networking websites is feasible and led to new HIV diagnosis [22]. The use of internet-based risk-reduction advice and treatment [23] could be offered to HSK users to minimise the potentially deleterious effects of reduced contact with clinical services. The optimal methods of kit design, distribution, and handling should be further refined with evaluation of different sites for distribution of HSK including a potential role for primary care services, pharmacies, and the internet. The cost-effectiveness of offering HSKs for HIV and STI testing via different sites should be assessed.

## Conclusions

Home sampling for STI and HIV offers an alternative to conventional STI clinic attendance for MSM seeking STI screening. It also significantly increases uptake of STI testing in HIV infected MSM. Home self-sampling could therefore be considered as an adjunct to conventional clinical services in order to improve STI/HIV testing outreach and uptake in this disproportionately affected group.
